# Giant 20/20 Meningioma: The Diagnostic Value of Confrontation Visual Fields

**DOI:** 10.7759/cureus.59754

**Published:** 2024-05-06

**Authors:** Sanjana Sundara Raj Sreenath, Gabriela Colina, Claudia M Prospero Ponce

**Affiliations:** 1 Department of Ophthalmology, Texas Tech University Health Sciences Center El Paso, El Paso, USA; 2 Department of Neurology, Texas Tech University Health Sciences Center El Paso, El Paso, USA; 3 Department of Ophthalmology and Neurology, Texas Tech University Health Sciences Center El Paso, El Paso, USA

**Keywords:** optic nerve atrophy, visual acuity, confrontation visual fields, giant meningioma, automated visual fields

## Abstract

Meningiomas are benign tumors of the central nervous system (CNS) that usually result in compression to adjacent structures and rarely cause pathology on their own. Meningiomas can affect the visual pathways originating from perineural or optic nerve sheath meningioma (ONSM), sellar, or clinoid, to the frontal-temporal-parietal-occipital lobes. Frontal meningiomas have an indolent presentation with frequent behavioral changes (i.e., personality or emotional changes, visual hallucinations), but they rarely present with visual disturbances. We present a case of a giant frontal meningioma causing progressive visual field loss despite preserved visual acuity and no behavioral changes. We aim to highlight the diagnostic value of performing a detailed ophthalmologic evaluation with confrontation visual field (CVF) testing and interpretation in aiding the discovery and diagnosis of intracranial tumors.

## Introduction

Meningiomas are central nervous system (CNS) tumors arising from the meningeal layer of the spinal cord or the brain. They are mostly noncancerous and slow-growing and account for a third of all primary CNS tumors [1]. However, based on their location and size, they can result in significant morbidity. While usually sporadic in nature, several risk factors such as obesity, exposure to ionizing radiation, hormone replacement therapy, breast cancer, and alcoholism have been documented [2,3]. The median age of diagnosis is 66 years, with a female-to-male ratio of 2.3:1 [1].

The most common locations for intracranial meningiomas are convexity, parasagittal, and falx, and less likely frontal [4]. Tumors in the frontal lobe generally affect cognitive and emotional functions and therefore may go unnoticed by the patient. These cases are typically referred to a psychiatrist initially. In this case, we present a patient with a giant frontal meningioma causing progressive visual field loss despite preserved visual acuity and no behavioral changes. 

## Case presentation

A 38-year-old previously healthy male sustained a motor vehicle accident after seeing an acute, chronic “yellow haze” while driving home. His vision had been progressively blurry over the previous two years. However, when he was seen by an optometrist, the patient was reassured that visual acuity was normal with a normal eye exam. Upon noticing acute worsening two months prior to admission, he consulted an ophthalmologist, who confirmed normal visual acuity but discovered an abnormal confrontation visual field (CVF) in both eyes. The patient then presented to our emergency department for evaluation. After thorough questioning, he endorsed right-sided headaches over a month, a 10-15 lb. weight loss over 3-4 months, decreased appetite, and anosmia for over two years. He denied hallucinations and any additional risk factors such as a history of radiation, hormonal intake, obesity, or family history. His wife denied any personality or behavioral changes. 

An ophthalmic exam revealed a visual acuity of 20/count fingers (CF) vision with pinhole improvement to 20/800 in the right eye (eccentric fixation) and 20/100 with pinhole improvement to 20/40 in the left eye. Physiologic anisocoria with the right pupil larger than the left was noted with no relative afferent pupillary defect (rAPD) evident. There was no red color desaturation, believed to be due to symmetry in optic nerve pallor. CVF testing revealed a central-cecocentral and superior scotoma in the right eye and macular-splitting temporal hemianopia in the left eye, indicative of a junctional scotoma of Traquair. Indirect ophthalmoscope fundoscopy showed a circumferential optic nerve pallor of 3+ in the right eye and temporal optic nerve pallor of 2+ in the left eye without edema and a normal cup-to-disk ratio. Cranial nerve testing 3-12 revealed no other abnormalities.

**Figure 1:**
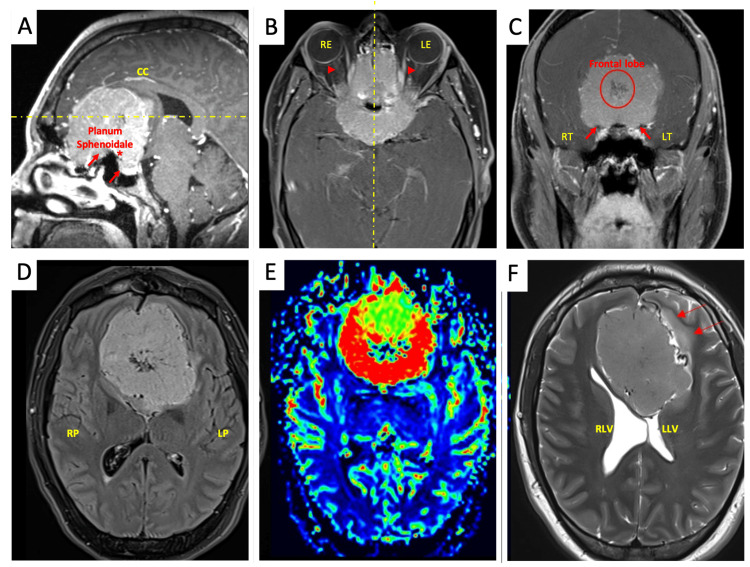
MRI brain with and without contrast RP/RT: Right parietal/temporal; LP/LT: left parietal/temporal; FLAIR: fluid-attenuated inversion recovery *Upper panel:* Sagittal (A), axial (B), and coronal (C) views showing a 6.1 x 6.5 x 4.8 cm interhemispheric parafalcine vascularized (circle) mass arising from the planum sphenoidale (short arrows) with significant mass effect to all anterior midline structures including the pituitary infundibulum (asterisk), suprasellar cistern, optic nerves (arrow head), right (RE) and left (LE) eyes, as well as optic chiasm (not shown). There is encasement of bilateral A1 and A2 segments of the anterior cerebral artery as well as supraclinoid segment of the bilateral internal carotid arteries (not shown). The frontal lobes are displaced superolaterally while displacing the corpus callosum (CC) and the anterior commissure posteriorly and superiorly (dotted yellow lines). *Lower panel*: Axial views show FLAIR (D), spectrometry (E), and T2 (F), highlighting the prominent internal vascularity of the tumor, and spectrometry demonstrates dense relative cerebral blood volume and hyperperfusion within the mass (red hue).There is a mass effect with enlargement of the right lateral ventricle (RLV) when compared to the left lateral ventricle (LLV) as well as the left frontal lobe vasogenic edema (red long arrows) where the mass effect is greater.

Brain MRI revealed a large 6.1 x 6.5 x 4.8 cm interhemispheric parafalcine mass arising from the planum sphenoidale with a significant mass effect on visual pathways (Figure 1). Surgical resection consisted of a two-phase surgery with the first stage involving a bifrontal craniectomy and tumor debulking via an anterior skull base approach with orbital arch bars, followed by orbital zygomatic arch bar osteotomies and bilateral decompression of the optic nerve.

**Figure 2:**
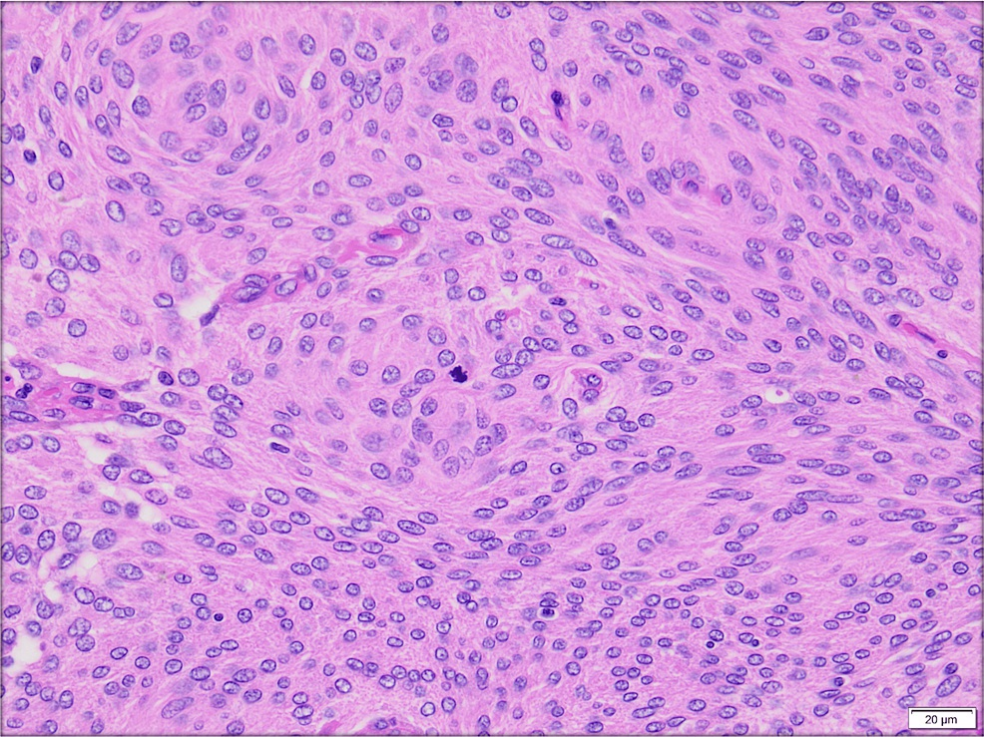
Hematoxilyn and eosin histology slide 200x depicting a meningioma Hematoxilyn and eosin histology slide 200x depicting a meningioma with mitotic count between 4 and 19 per 10 high-power field, confirming the diagnosis of atypical meningioma Grade II. The highlighted red circle shows a mitosis. Acknowledgment: Dr. Jonathan Lavezo, MD, NeuroPathology specialist, TTUHSC at El Paso/UMC Pathology Department.

A pathology report confirmed a Grade II, atypical meningioma (Figure 2). Interestingly, after his second surgery, he reported increased appetite, improved energy, clearer vision while watching TV, and no longer required his glasses to see. Postoperative MRI brain showed significant tumor debulking and decompression of the optic nerves (leading to improvement in visual acuity) and chiasm with resolution of mass effect on adjacent structures. 

**Figure 3:**
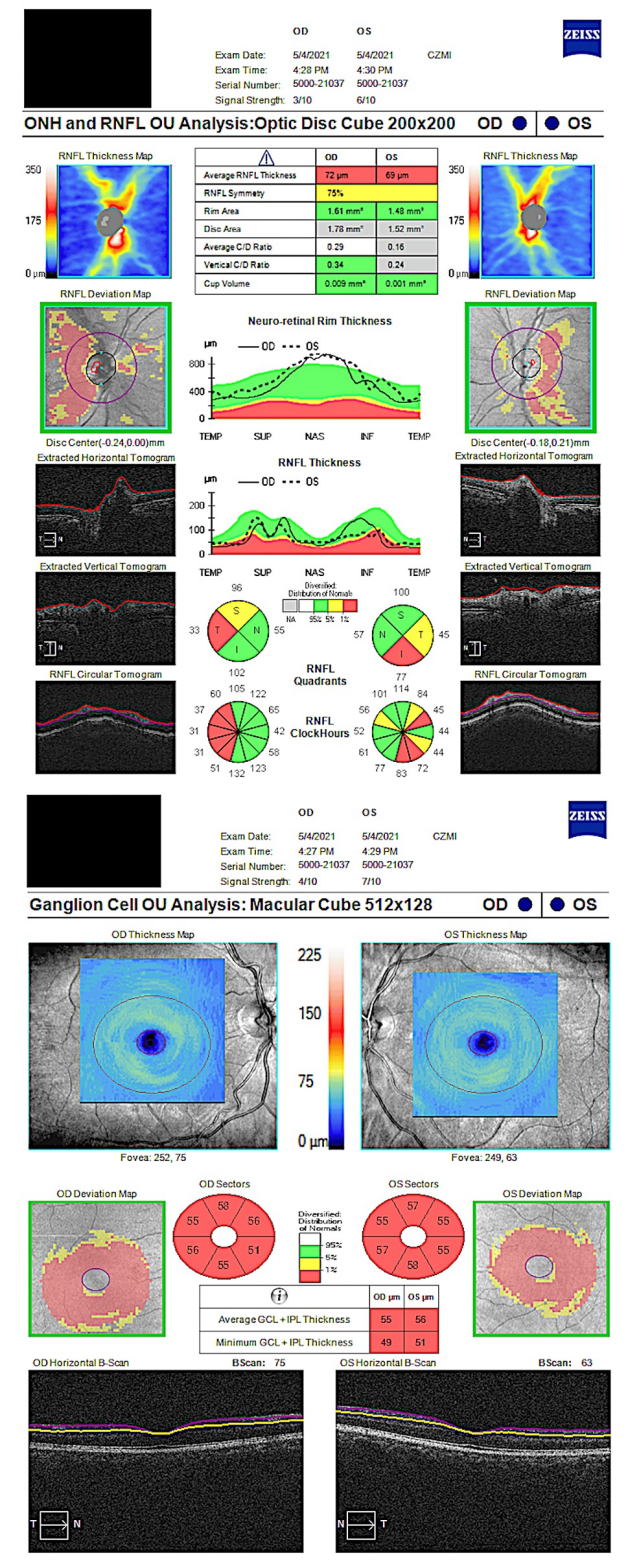
Optical coherence tomography (OCT) showing bilateral optic nerve atrophy OCT showing bilateral optic nerve atrophy. Retinal nerve fiber layer (RNFL) (A) confirmed the permanent diffuse thinning bilaterally predominantly on the superior and inferior quadrants, with an average thickness of 72 um in the right eye (OD) and 69 um in the left eye (OS) (normal 100-120 um) and ganglion cell layer (B) atrophy of <60 um in both eyes (normal > 80 um).

**Figure 4:**
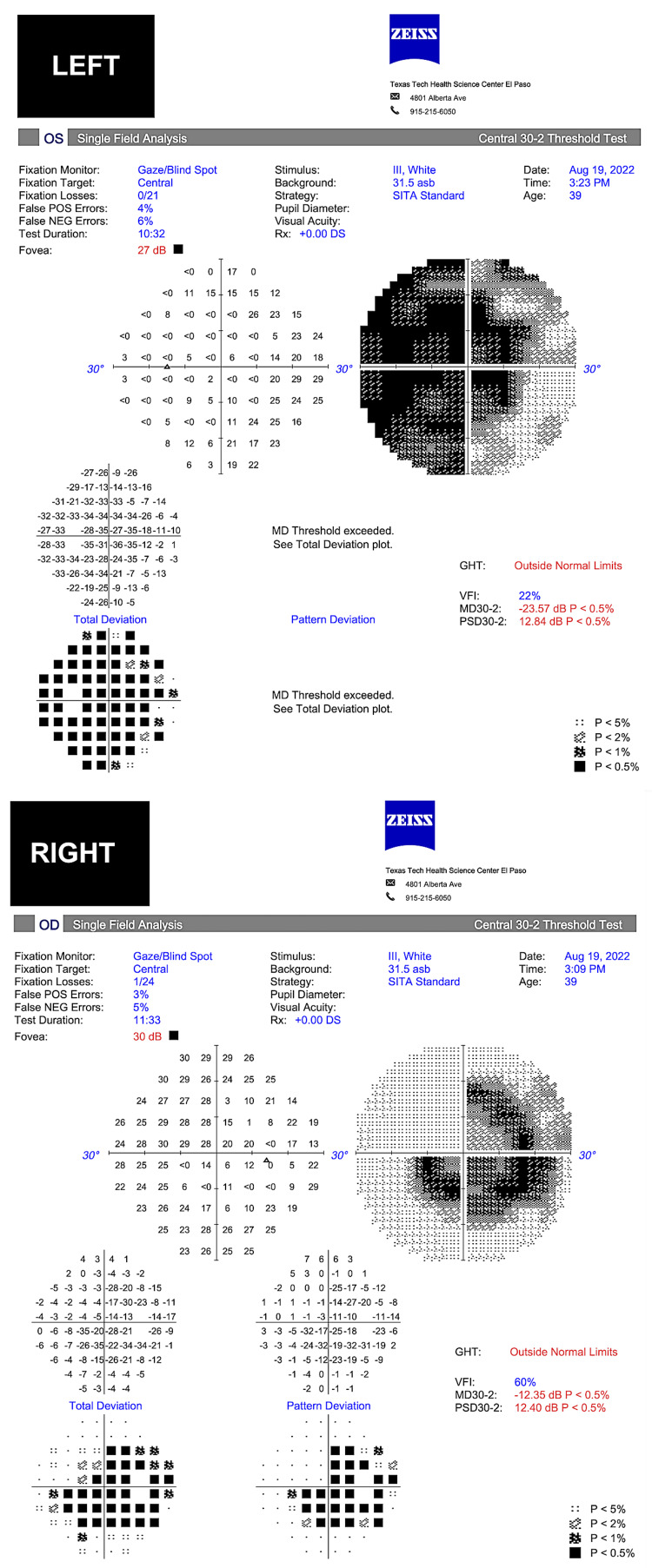
Automated visual field testing Automated Humphrey visual field 30-2, stimulus III, shows a mild decreased fovea sensitivity in the right eye and an incomplete central and paracentral scotoma with normal far periphery, while the left eye visual field testing shows moderate decreased fovea sensitivity, dense central scotoma, almost complete temporal hemianopia, and some sparing of far superior and inferior peripheral nasal field. Both show good reliability. Reliability tests have a false-positive and false-negative rate below 33% as well as a below 20% fixation loss, per manufacturer.

Despite persistent bilateral optic nerve atrophy and pallor (Figure 3), his final uncorrected visual acuity was 20/20 in both eyes. Final automated visual field testing showed permanent central-cecocentral scotoma in the right eye and diffuse constriction in the left eye (Figure 4). Functional CVF testing was improved to finger counting accurately in all quadrants. His visual function has remained stable for up to a year.

## Discussion

Meningiomas are the most common primary benign brain tumor in adults. Diagnosis is usually made through an MRI or a contrast-enhanced CT. However, if visual pathways are affected, a detailed ophthalmologic examination including visual acuity, CVF testing, and the presence of rAPD is vital. Additionally, the red-color desaturation testing, which tests for color density difference between the eyes, may indicate decreased optic nerve function in one eye more than the other, as long as it is not bilateral as in our case.

**Figure 5:**
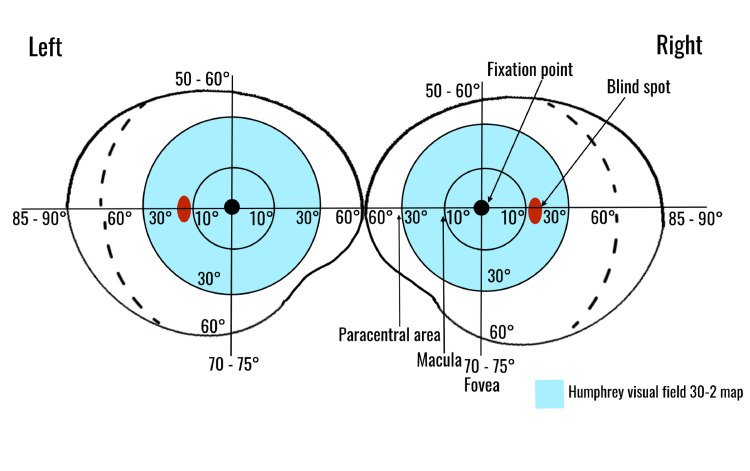
Depiction of human visual field degrees Confrontation visual fields (CVFs) allow all quadrants to be tested (full extension) as long as the target is placed accurately and symmetrically. Automated visual fields, on the other hand, can only extend to 30° from the center/fovea to either side (highlighted in blue). Temporally, the visual field reaches 90° from the point of central fixation and 60° nasally as represented by the dashed line. Image credits: Sanjana Sundara Raj Sreenath

Visual field defects are frequently highly localizable for a lesion in the visual pathway. The initial, broader approach to test visual fields is by confrontation (Figure 5) since one can evaluate the full extension.

Treatment for meningiomas is patient-specific but could involve observation for small, asymptomatic tumors and radiotherapy or surgical resection for large or symptomatic meningiomas. Depending on pathologic behavior/grade, adjuvant treatment may be needed. The World Health Organization (WHO) classifies meningiomas into three grades based on histopathological characteristics [5]. Grade 1, which comprises more than 80% of all meningiomas, includes benign findings without anaplastic features. Grade 2 meningiomas are characterized as having between four and 19 mitoses per ten high power fields and brain infiltration and can be of the choroid or clear cell subtypes. Grade 3 consists of malignant or anaplastic lesions with more than 20 mitoses per ten high power fields. Our patient had a Grade 2 atypical meningioma arising from the planum sphenoidale extending to the interhemispheric parafalcine regions bilaterally [6,7].

Planum sphenoidale meningiomas represent about 10% of the intracranial meningiomas. Because of the slow growth of the tumor, these meningiomas are usually discovered when they reach large sizes (>6cm), similar to the dimensions observed in our case report. Headache, anosmia, personality changes, visual impairment, increased intracranial pressure, and seizures are the most common presenting symptoms of planum sphenoidale meningiomas [8]. Our patient did have anosmia but was not concerned about it. There were no personality changes according to the wife. Because near-total tumor resection was achieved, no adjuvant treatment was needed up to one year postop.

**Figure 6:**
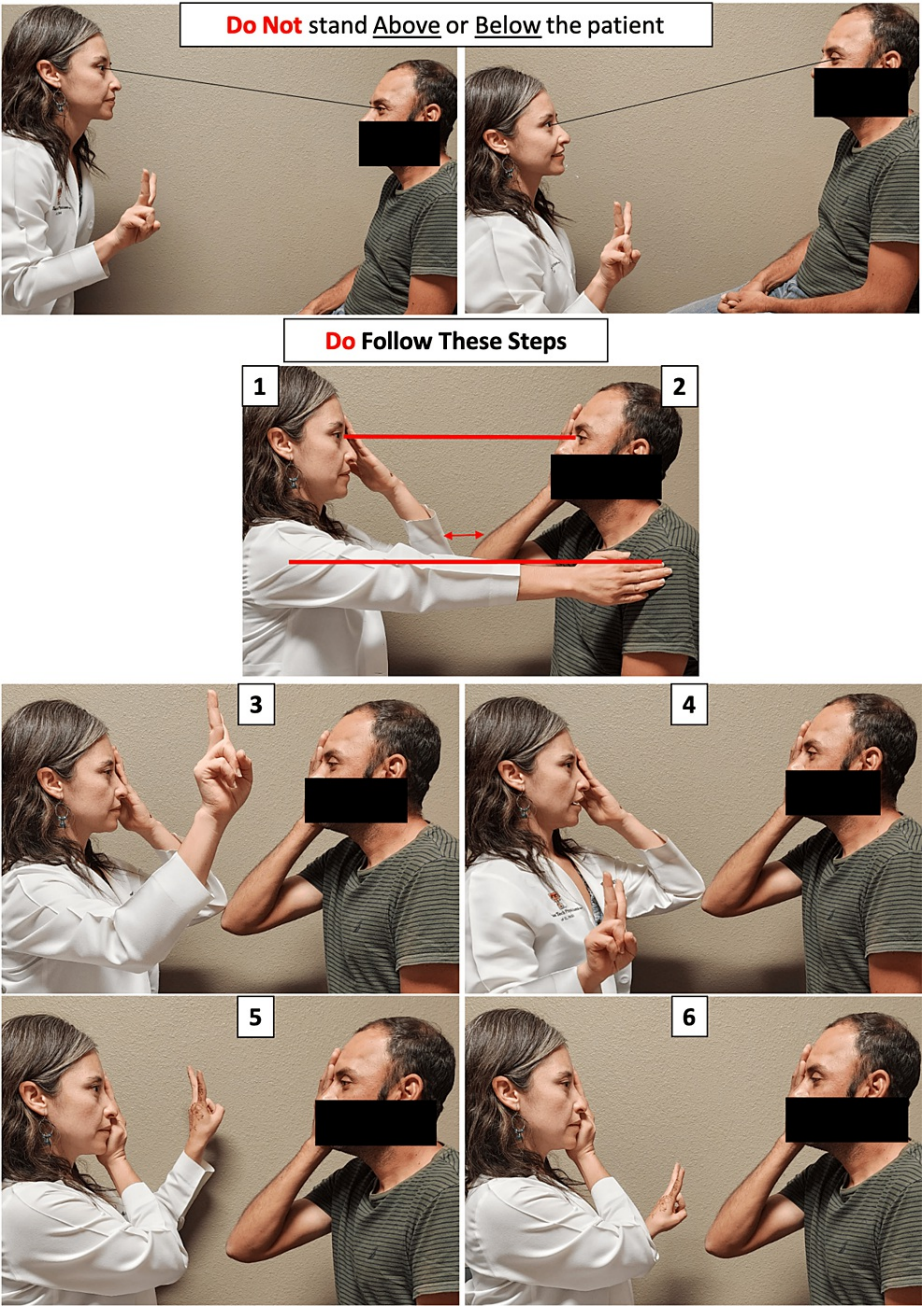
Technique for confrontation visual field (CVF) testing The examiner is seated at arm’s length in front of the patient (1) with eyes in straight alignment (2). The patient is asked to cover one eye, and the examiner covers the eye in front. One, two, or five fingers are held halfway between the patient and the examiner in one of the four quadrants. The patient is asked to count the number of fingers. Once all four quadrants are tested, the examiner switches to the patient’s other eye. Written informed consent has been obtained from the patient.

Visual symptoms such as bitemporal hemianopia (due to optic chiasm compression) may progress relatively unnoticed by the patient, due to the slow growth [8]. A patient may not become fully concerned about these symptoms until a significant event occurs, such as the motor vehicle accident or an external inciting trauma brought on by bumping into walls or door frames, for instance. Visual acuity may be decreased with tumor growth but is neither a sensitive nor an early sign of optic nerve compromise [8]. Our patient had consulted several eye care providers before but was informed that his exam was normal because of “normal visual acuity.” CVF testing is, therefore, a very important diagnostic tool in examining visual function, and it must be accurately performed (Figure 6). Fortunately for this patient, visual acuity improved from 20/800 in the right eye and 20/40 in the left eye to 20/20 in both eyes after tumor removal.

## Conclusions

Giant planum sphenoidale meningiomas are rare but are known for their variability of symptom presentations. Here, we present a patient with a frontal meningioma causing progressive visual field loss but preserved visual acuity. Due to the location and slow growth of frontal meningiomas, the only initial finding might be an abnormal confrontation field test and normal visual acuity, as in the present case. Visual field defects are highly localizing signs of brain pathology. Therefore, through this case, we aim to highlight the diagnostic value of confirmatory automated visual field testing. Enquiring about red flag symptoms such as anosmia, headaches, and personality/behavior may also allow for quicker recognition of an often-incidental diagnosis.
